# Design and Synthesis of Novel Pyrazole-Substituted Different Nitrogenous Heterocyclic Ring Systems as Potential Anti-Inflammatory Agents

**DOI:** 10.3390/molecules22040512

**Published:** 2017-03-24

**Authors:** Eman S. Nossier, Hoda H. Fahmy, Nagy M. Khalifa, Wafaa I. El-Eraky, Marawan A. Baset

**Affiliations:** 1 Department of Pharmaceutical Chemistry, Faculty of Pharmacy (Girls), Al-Azhar University, Cairo 11754, Egypt; emy28_s@hotmail.com; 2 Department of Therapeutical Chemistry, Pharmaceutical and Drug Industries Division, National Research Centre, Giza 12622, Egypt; hh_fahmy@yahoo.com; 3 Pharmaceutical Chemistry Department, Drug Exploration & Development Chair (DEDC), College of Pharmacy, King Saud University, Riyadh 11451, Saudi Arabia; 4 Pharmacology Department, National Research Centre, Dokki, Cairo 12622, Egypt; wafaa_nrc@yahoo.com (W.I.E.-E.); dr.marawan@gmail.com (M.A.B.)

**Keywords:** 1,3-diaryl pyrazole derivatives, anti-inflammatory activity, synthesis

## Abstract

With the aim of developing novel anti-inflammatory scaffolds, a new series of pyrazole-substituted various nitrogenous heterocyclic ring systems at C-4 position were synthesized through different chemical reactions and validated by means of spectral and elemental data. The new obtained compounds were investigated for their anti-inflammatory activity using the carrageenan-induced paw edema standard technique and revealed that, compound **6b** showed increased potency with % inhibition of edema 85.23 ± 1.92 and 85.78 ± 0.99, respectively, higher than the standard reference drugs indomethacin and celebrex (72.99% and 83.76%). Molecular modeling studies were initiated herein to validate the attained pharmacological data and provide understandable evidence for the observed anti-inflammatory behavior.

## 1. Introduction

**Figure 1:**
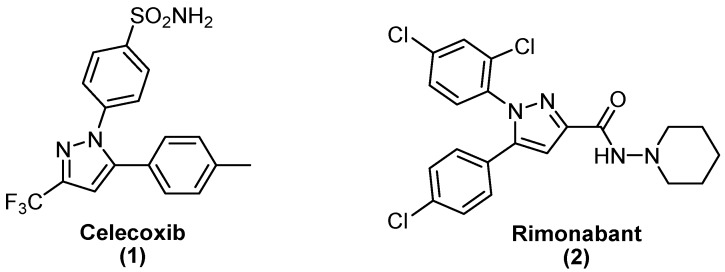
Structures of the selective COX-2 inhibitors, celecoxib and Rimonabant.

The effective and quick preparation of biologically active compounds has encouraged Researchers to identify new strategies which could be beneficial to the pharmaceutical industry. Pyrazole analogs are a class of bioactive nitrogenous heterocycles, playing an essential role in the medicinal chemistry fields. Incorporation of different aryl and sulphonamides onto pyrazole nucleus have resulted in Celecoxib (**1**) and Rimonabant (**2**) which are anti-inflammatory drugs (Figure 1). Recently, Alegaon et al. in 2014 have reported some 1,3,4-trisubstituted pyrazole derivatives are potent anti-inflammatory activity and COX-2 selective inhibition [1,2]. In addition, numerous reports have appeared in the literature describing different bioactivities and good safety profiles of 1,3,4-trisubstituted pyrazole derivatives including: anti-inflammatory [3,4,5,6,7], analgesic, lipid peroxidation, ulcerogenic [8,9], antipyretic [10], antioxidant [11], antimicrobial, antiviral [12,13,14], anticancer [15,16,17,18], antimitotic [19], and immunosuppressive agents [20]. In addition, some pyrazole compounds have gained great attention as antibacterial and fungicidal isoforms of human cytosolic carbonic anhydrase I or II and antitumor properties [21,22,23]. Extension of our research towards the identification of an efficient synthesis of biologically active pyrazole compounds [24,25,26,27,28,29], we report herein the synthesis of novel derivatives of 1,3-diaryl pyrazoles and their anti-inflammatory activities.

## 2. Results and Discussion

### 2.1. Chemistry

**Scheme 1:**
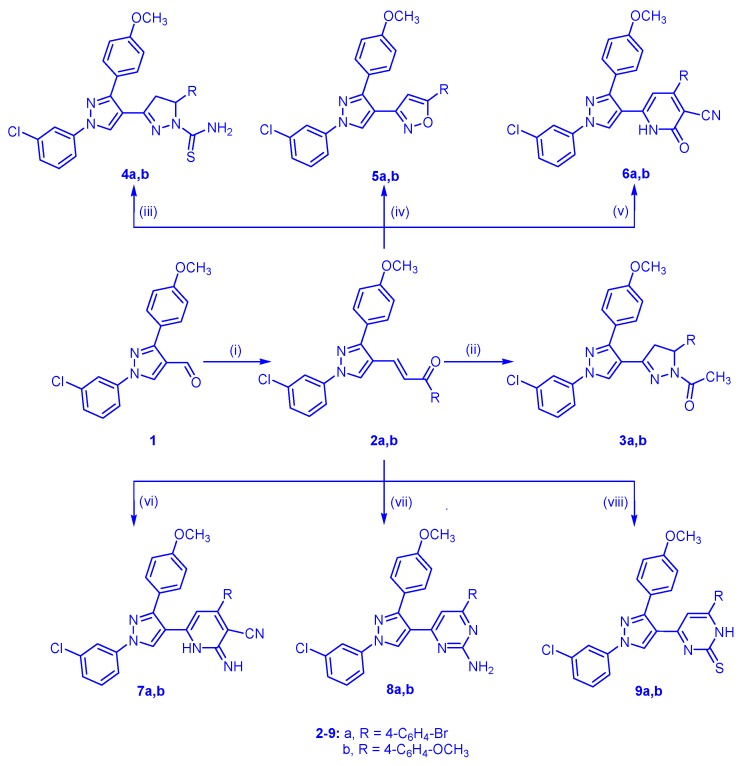
Synthetic route for polysubstituted pyrazole compounds **2**–**9**. Reagents and Conditions: (**i**) RCOCH_3_ derivatives/30%Etanolic NaOH/r.t./12 h, 83%–85%; (**ii**) N_2_H_4_·H_2_O/AcOH/reflux/4–6 h, 73%–81%; (**iii**) NH_2_CSNHNH_2_/EtOH/NaOH/reflux/2–3 h, 71%–73%; (**iv**) NH_2_OH·HCl/ethanol/NaOH/reflux/2–3 h, 80%–85%; (**v**) NC-CH_2_COOEt/CH_3_COONH_4_^+^/ethanol//reflux/6 h, 75%–76%; (**vi**) CNCH_2_CN/CH_3_COONH_4_^+^/ethanol/reflux/6 h, 70%–73%; (**vii**) NH_2_NHNH_2_.H_2_SO_4_/ethanol/NaOH/reflux/5–7 h, 69%–71%; (**viii**) NH_2_CSNH_2_/ethanol/NaOH/reflux/6–8 h, 80%–81%.

The reaction sequences outlined in Scheme 1 was used for the synthesis of the target compounds. Application of the Claisen Schmidt condensation on substituted acetophenones namely, 4-bromoacetophenone or 4-methoxyacetophenone and 1-(3-chlorophenyl)-3-(4-methoxyphenyl)-1*H*-pyrazole-4-carboxaldehyde (**1**) in ethanolic sodium hydroxide solution afforded (*E*)-1-(4-substituted phenyl)-3-(1-(3-chlorophenyl)-3-(4-methoxyphenyl)-1*H*-pyrazol-4-yl)prop-2-en-1-one (**2a**,**b**), which was used as starting materials. Cyclocondensation of the α,β-unsaturated ketone **2a**,**b** with hydrazine hydrate in glacial acetic acid yielded the corresponding pyrazoline derivatives **3a**,**b**. On the other hand, heating of **2a**,**b** with thiosemicarbazide in ethanolic NaOH provided 1-thiocarbamoyl pyrazole derivatives **4a**,**b**. In addition, condensation of compound **2a**,**b** with hydroxylamine hydrochloride in refluxing ethanol in the presence of sodium hydroxide as alkaline medium afforded the corresponding isoxazoline **5a**,**b**. Reaction of α,β-unsaturated ketone **2a**,**b** with ethyl cyanoacetate, or malononitrile in presence of ammonium acetate, gave the corresponding 2-oxo(imino)pyridine derivatives **6a**,**b**, and **7a**,**b**, respectively. Furthermore, treatment of **2a**,**b** with guanidine sulfate in ethanolic sodium hydroxide gave 2-aminopyrimidine derivatives **8a**,**b**. Finally, treatment of **2a**,**b** with thiourea in presence of sodium hydroxide gave the corresponding pyrimidine-2-thione derivative **9a**,**b** (Scheme 1).

### 2.2. Biological Evaluation

#### 2.2.1. In Vivo Anti-Inflammatory Activity

**Figure 2:**
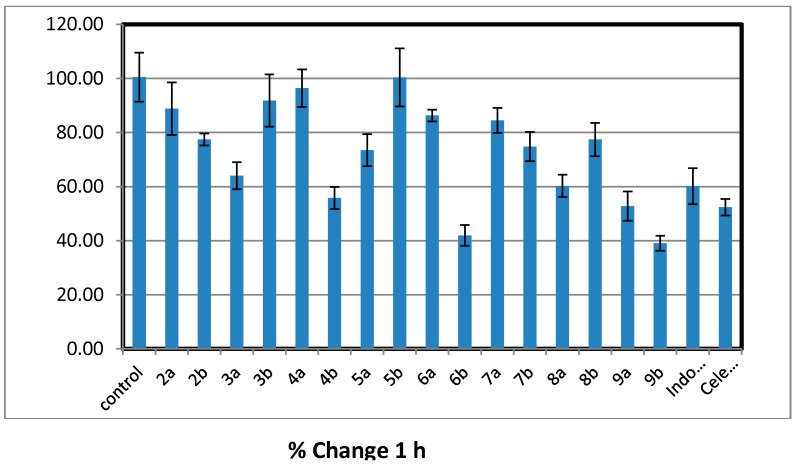
Percentage of inhibition after 1 h of carrageenan injection comparable to that of standard drugs, indomethacin and Celebrex.

**Figure 3:**
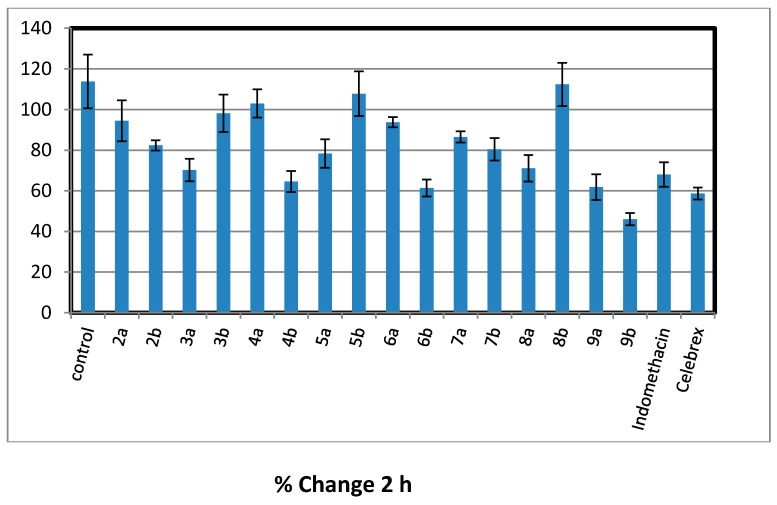
Percentage of inhibition after 2 h of carrageenan injection comparable to that of standard drugs, indomethacin and Celebrex.

**Figure 4:**
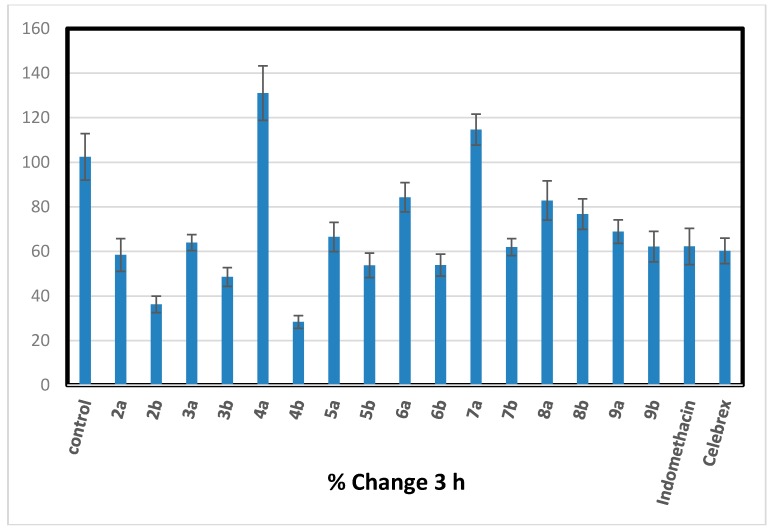
Percentage of inhibition after 3 h of carrageenan injection comparable to that of standard drugs, indomethacin and Celebrex.

**Figure 5:**
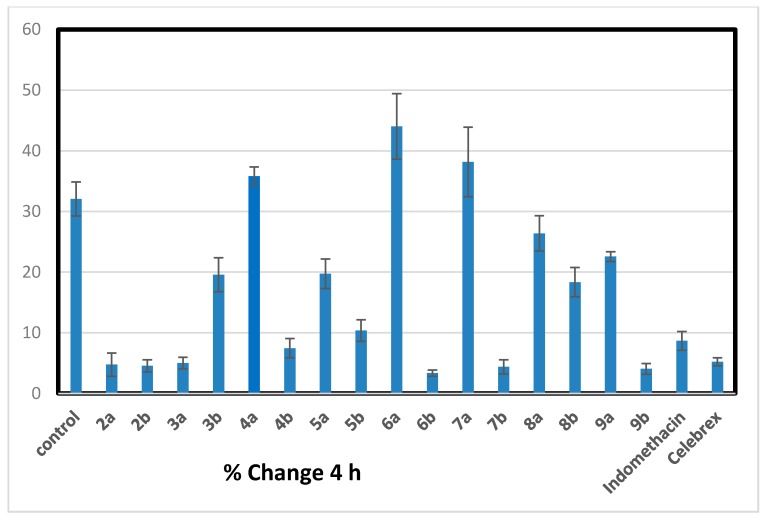
Percentage of inhibition after 4 h of carrageenan injection comparable to that of standard drugs, indomethacin and Celebrex.

All the newly synthesized trisubstitutedpyrazole compounds **2**–**9** were evaluated for their in vivo anti-inflammatory activity using carrageenan induced rat paw edema method [30]. The primarily antiinflammatory activity results (Table 1) revealed that, six compounds (**2a**, **2b**, **3a**, **6b**, **7b**, and **9b**) showed consistently excellent anti-inflammatory activity (84.39%–89.57% inhibition) 4 h after the carrageenan injection comparable to that of standard drugs indomethacin and celebrex (72.99% and 83.76%), respectively. Chalcones **2a** and **2b** showed approximately equal anti-inflammatory activity higher than the reference drugs (% inhibition of edema = 85.23 ± 1.92 and 85.78 ± 0.99), respectively. Considering of the target pyrazoles, it was noticed that the electron donating substituent (methoxy group) at position-4 of pyrazole moiety exhibited higher activity than their congeners with 4-electron withdrawing substituent (bromo group) except for acetylpyrazoline derivatives **3a**,**b**. Cyclization of α,β-unsaturated ketone **2b** bearing 4-methoxyphenyl at position-4 of pyrazole moiety, gave increased activity in compounds (**6b**, **7b**, and **9b**) with little decrease in acetyl pyrazoline derivative **3b** and marked decrease in compounds (**4b**, **5b**, and **8b**). However, cyclization of chalcone **2a** having 4-bromophenyl at position-4 of pyrazole moiety led to a drop in anti-inflammatory activity as in **3a**–**9a** derivatives. The cyanopyridone derivative **6b** seems to be the most effective prepared anti-inflammatory agent, revealing better activity (89.57% inhibition of edema) than both indomethacin and celecoxib (standard drugs). Insertion of cyanoiminopyridine moiety as in **7a**,**b** (19.11% and 86.37% inhibition) instead of cyanopyridone in **6a**,**b** (37.35% and 89.57 % inhibition) displayed a little decrease in the activity. While replacement of thiopyrimidine in **9a**,**b** (29.61% and 87.42% inhibition) with aminopyrimidine moiety in **8a**,**b** led to a drop in the activity (17.66% and 42.78% inhibition) (Figure 2, Figure 3, Figure 4 and Figure 5).

#### 2.2.2. Ulcerogenic Liability

Ulcerogenic liability of all prepared anti-inflammatory agents **2**–**9** was determined following the reported standard method [31] using indomethacin and Celebrex (in a dose 0.28 mmol/kg) as reference standards. It was noticed that all compounds revealed no ulcers, like celebrex, and they are considered safer than indomethacin itself which produced an ulcer count of 14 ± 1.2.

### 2.3. Molecular Modeling Study

Molecular modeling study of the highly observed anti-inflammatory active agent **6b** was performed herein to understand the observed pharmacological data. Docking study was initiated using MOE 2008.10 program. It is used to predict the binding modes and orientation of compound **6b** at the active site of the ATP binding site of COX-2 enzyme. The coordinates of this enzyme structure were obtained from the crystal structure of COX-2 with its inhibitor (PDB ID: 1CX2). The root mean square difference (RMSD) between the top docking pose and original crystallographic geometry of co-crystallized ligand SC-558 was 0.9 Å. The phenylsulphonamide moiety of this SC-558 is surrounded by hydrophobic residues Leu352, Tyr355, Phe518, Val523, and the backbone of Ser353. Beyond this hydrophobic pocket, the sulphonamide exhibits hydrophilic interaction with His90, GIn192, and Arg513 [32]. Celecoxib forms three hydrogen bonds with the hydrophilic side chains (His90 and Gln192) in the side pocket and the main chain carbonyl at residue Leu338 [33].

In Table 2, the compounds **2a**, **2b**, **3a**, **4b**, **5b**, **6b**, **7b** and **9b** with the highest and moderate anti-inflammatory activity were found to have high binding energy ranging from −6.25 to −8.11 kcal·mol^−1^ in comparison with reference ligands SC-558 and celecoxib (−6.30 and −6.55 kcal·mol^−1^ respectively). It was observed that most of the active compounds have arene-cation interaction between (Arg120 and Arg513) and the newly inserted 4-methoxyphenyl moiety.

**Figure 6:**
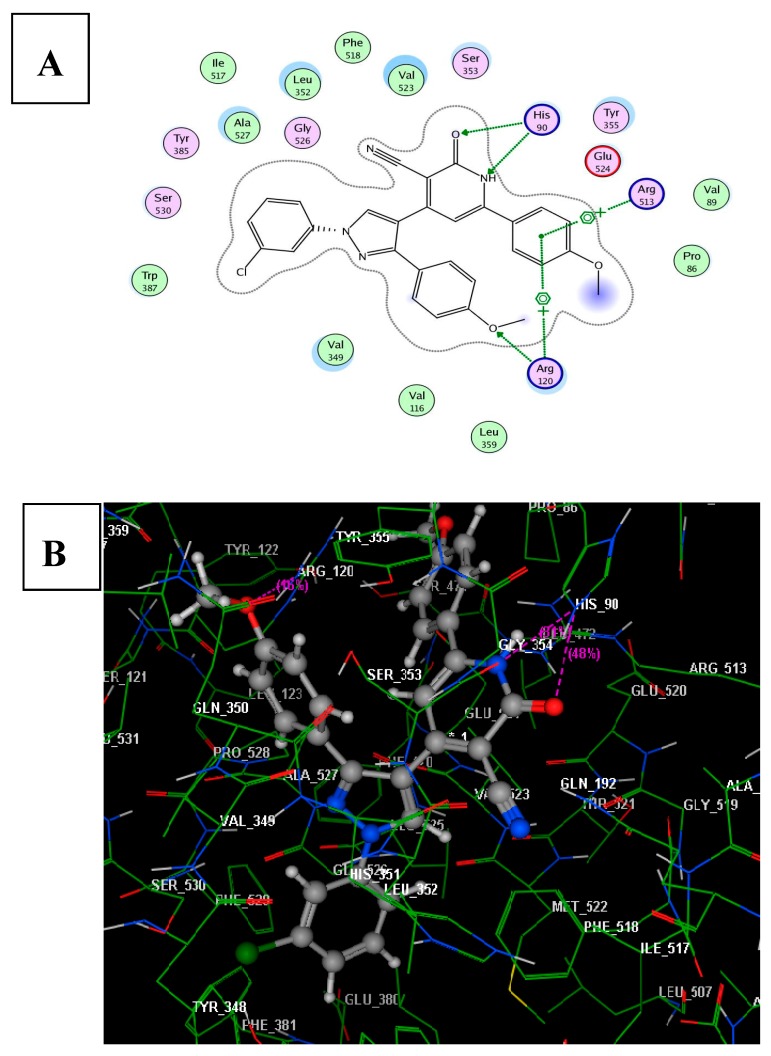
The proposed binding mode of compound **6b** docked in the active site of COX-2; (**A**,**B**) showing 2D and 3D ligand-receptor interactions (hydrogen bonds are illustrated as dotted purple lines; C atoms are colored gray, N blue and O red).

From Figure 6, it was found that the compound **6b** revealed good fitting inside the binding site of the protein molecular surface and having minimum binding energy of −8.11 kcal·mol^−1^. There were two hydrogen bonds linking the sidechain of His90 with NH and oxygen of pyridone moiety as hydrogen acceptors (distance: 2.76 and 2.79 Å, respectively). The 4-methoxyphenyl moiety attached to pyridone formed two arene-cation interactions with Arg120 and Arg513. Furthermore, one H-bond acceptor was observed between the sidechain of Arg120 and 4-methoxyphenyl linked to pyrazole scaffold (distance: 3.19 Å). The previous results indicated that the insertion of 4-methoxyphenyl group to the pyridone moiety might reinforce the combination of compound **6b** and the receptor, which might enhance the binding affinity, resulting in the increased anti-inflammatory activity of this compound.

## 3. Experimental Section

### 3.1. General Information

Melting points were measured in open capillary tubes using a Griffin apparatus and are uncorrected. Structures of compounds were confirmed by routine spectrometric analysis. Elemental analyses were carried results were within ±0.4% of the theoretical values. Infrared spectra were recorded on a 435 IR spectrophotometer (Shimadzu Bruker, Tokyo, Japan) using KBr discs. ^1^H-NMR and ^13^C-NMR spectra were obtained on a Gemini 500 MHz spectrophotometer (Varian, Polo Alto, CA, USA) or on a Bruker 500 MHz spectrophotometer, and measured in δ scale using TMS as an internal standard. Mass Spectra were recorded on a 5988 spectrometer (Hewlett Packard, Palo Alto, CA, USA). Analytical thin layer chromatography (TLC) was performed using silica gel aluminum sheets, 60 F_254_ (E. Merck, Darmstadt, Germany) for the progress of reactions and visualization with ultraviolet light (UV) at 365 and 254 nm.

#### 3.1.1. 1-(4-Substitutedphenyl)-3-(1-(3-chlorophenyl)-3-(4-methoxyphenyl)-1*H*-pyrazol-4-yl)prop-2-en-1-one (**2a**,**b**)

A mixture of pyrazolecarbaldehyde derivative **1** (0.01 mol) and substituted acetophenones, namely 4-bromoacetophenone or 4-methoxyacetophenone (0.01 mol), in of 30% ethanolic NaOH solution (40 mL) was stirred for 12 h at room temperature. The progress of reaction was monitored by TLC. After completion, the reaction mixture was poured into acidified ice cold water of pH ~2. The precipitated solid formed was filtered, washed with water and recrystallized from ethanol to afford the title compounds **2a**,**b**.

*1-(4-Bromophenyl)-3-(1-(3-chlorophenyl)-3-(4-methoxyphenyl)-1H-pyrazol-4-yl)prop-2-en-1-one* (**2a**). Yield 83%; m.p. 235–237 °C; IR (KBr, cm^−1^) ν: 1673 (C=O), 1589 (C=C); ^1^H-NMR (DMSO-*d*_6_-δ ppm): 3.81 (s, 3H, OCH_3_); 7.09–7.99 (m, 14H, ArH + CH=CH), 9.45 (s,1H, CH of pyrazole); ^13^C-NMR (DMSO-*d*_6_-δ ppm): 55.60, 114.34, 116.62, 117.75, 125.11, 125.95, 127.67, 129.08, 129.81, 131.74, 133.85, 134.47, 135.44, 141.08, 144.32, 151.54, 159.58, 191.96; MS (EI, 70 eV): *m*/*z* (%): 492 (11) [M]^+^; Anal. Calcd. for C_25_H_18_BrClN_2_O_2_ (493.78): C, 60.81; H, 3.67; N, 5.67; Found: C, 61.65; H, 3.77; N, 5.32.

*1-(4-Methoxphenyl)-3-(1-(3-chlorophenyl)-3-(4-methoxyphenyl)-1H-pyrazol-4-yl)prop-2-en-1-one* (**2b**). Yield 85%; m.p. 152–154 °C; IR (KBr, cm^−1^) ν: 1680 (C=O), 1601 (C=C); ^1^H-NMR (DMSO-*d*_6_-δ ppm): 3.80, 3.85 (2s, 6H, 2OCH_3_); 7.10–8.09 (m, 14H, ArH + CH=CH), 9.43 (s,1H, CH of pyrazole); MS (EI, 70 eV): *m*/*z* (%): 447 (11, M^+^ + 3), 445 (29, M^+^ + 1), 410 (100); Anal. Calcd. for C_26_H_21_ClN_2_O_3_ (444.91): C, 70.19; H, 4.76; N, 6.30; Found: C, 70.35; H, 4.52; N, 6.55.

#### 3.1.2. 1-(3-(4-Substituted phenyl)-5-(1-(3-chlorophenyl)-3-(4-methoxyphenyl)-1*H*-pyrazol-4-yl)-4,5-dihydro-pyrazol-1-yl ethanone (**3a**,**b**)

A solution of hydrazine hydrate (0.01 mol) was added to a solution of compounds **2a**,**b** (0.01 mol) in glacial acetic acid (20 mL) and the mixture was refluxed for 4–6 h. The reaction mixture was cooled to room temperature and the precipitated solid was filtered, washed with water, dried, and recrystallized from ethanol to give the title compounds **3a**,**b**.

*1-(3-(4-Bromophenyl)-5-(1-(3-chlorophenyl)-3-(4-methoxyphenyl)-1H-pyrazol-4-yl)-4,5-dihydro-pyrazol-1-yl ethanone* (**3a**). Yield 73%; m.p. 233–235 °C; ^1^H-NMR (DMSO-*d*_6_-δ ppm): 2.28 (s, 3H, COCH_3_), 3.16 (dd, 1H, CH), 3.77 (s, 3H, OCH_3_), 3.80 (dd, 1H, CH), 5.63 (dd, 1H, CH), 7.01–8.43 (m, 12H, Ar-H), 9.68 (s, 1H, CH-pyrazole); ^13^C-NMR (DMSO-*d*_6_-δ ppm): 24.0, 43.8, 55.6, 63.9, 114.3, 116.8, 117.6, 125.4, 125.9, 126.1, 127.6, 129.1, 129.8, 130.7, 131.1, 134.4, 137.8, 140.8, 150.4, 155.2, 159.1, 160.9, 168.2; MS (EI, 70 eV): *m*/*z* (%): 548 (43) [M]^+^; Anal. Calcd. for C_27_H_22_BrClN_4_O_2_ (549.85): C, 58.98; H, 4.03; N, 10.19; Found: C, 58.77; H, 4.37; N, 10.36.

*1-(3-(4-Methoxyphenyl)-5-(1-(3-chlorophenyl)-3-(4-methoxyphenyl)-1H-pyrazol-4-yl)-4,5-dihydro-pyrazol-1-yl ethanone* (**3b**). Yield 81%; m.p. 175–179 °C; ^1^H-NMR (DMSO-*d*_6_-δ ppm): 2.27 (s, 3H, COCH_3_), 3.13 (dd, 1H, CH), 3.76, 3.77 (2s, 6H, 2OCH_3_), 3.80 (dd, 1H, CH), 5.59 (dd, 1H, CH), 6.98–8.87 (m, 12H, Ar-H), 9.69 (s, 1H, CH-pyrazole) ppm; ^13^C-NMR (DMSO-*d*_6_-δ ppm): 24.01, 43.8, 55.6, 63.9, 114.3, 116.8, 117.6, 125.4, 125.9, 126.1, 127.6, 129.1, 129.8, 130.7, 131.1, 134.4, 137.8, 140.8, 150.4, 155.2, 159.1, 160.9, 168.2 ppm; MS (EI, 70 eV): *m*/*z* (%): 503 (11, M^+^ + 3), 501 (25, M^+^ + 1), 77 (100); Anal. Calcd. for C_28_H_25_ClN_4_O_3_ (500.98): C, 67.13; H, 5.03; N, 11.18; Found: C, 67.38; H, 4.95; N, 11.13.

#### 3.1.3. 3-(4-Substitutedphenyl)-5-(1-(3-chlorophenyl)-3-(4-methoxyphenyl)-1*H*-pyrazol-4-yl)-4,5-di-hydropyrazole-1-carbothioamide (**4a**,**b**)

Thiosemicarbazide was added to a mixture of chalcone **2a**,**b** (0.01 mol) in absolute ethanol (30 mL) containing sodium hydroxide (1 g, 0.025 mol). The reaction mixture was heated under reflux for 2–3 h. The contents were reduced, cooled, and poured onto crushed ice containing a few drops of hydrochloric acid (until pH ~6). The resulting precipitate was collected by filtration and recrystallized from methanol to give the title compounds **4a**,**b**.

*3-(4-Bromophenyl)-5-(1-(3-chlorophenyl)-3-(4-methoxyphenyl)-1H-pyrazol-4-yl)-4,5-di-hydropyrazole-1-carbothioamide* (**4a**). Yield 71%; m.p. 284–286 °C; IR (KBr, cm^−1^) ν: 3414 (NH_2_), 1592 (C=C), 1067 (C=S); ^1^H-NMR (DMSO-*d*_6_-δ ppm): 3.24 (dd, 1H, CH), 3.78 (s, 3H, OCH_3_), 3.81 (dd, 1H, CH), 6.00 (dd, 1H, CH), 7.00–8.25 (m, 14H, Ar-H + NH_2_ exchangeable with D_2_O), 9.17 (s, 1H, CH of pyrazole); ^13^C-NMR (DMSO-*d*_6_-δ ppm): 40.5, 55.8, 56.2, 114.5, 114.7, 116.9, 118.0, 118.5, 124.3, 125.6, 126.1, 129.6, 129.9, 130.9, 131.6, 132.0, 134.4, 141.0, 150.1, 154.1, 159.7, 176.5; MS (EI, 70 eV): *m*/*z* (%): 569 (1, M + 4), 567 (2.7, M + 2), 565 (2.7, M^+^), 111 (100); Anal. Calcd. for C_26_H_21_BrClN_5_OS (566.9): C, 55.09; H, 3.73; N, 12.35; Found: C, 54.92; H, 3.52; N, 12.14.

*3-(4-Methoxyphenyl)-5-(1-(3-chlorophenyl)-3-(4-methoxyphenyl)-1H-pyrazol-4-yl)-4,5-dihydro-pyrazole-1-carbothioamide* (**4b**). Yield 73%; m.p. 223–225 °C; IR (KBr, cm^−1^) ν: 3444 (NH_2_), 1594 (C=C), 1092 (C=S); ^1^H-NMR (DMSO-*d*_6_-δ ppm): 3.24 (dd, 1H, CH), 3.75, 3.78 (2s, 6H, 2OCH_3_), 3.84 (dd, 1H, CH), 5.98 (dd, 1H, CH), 6.95–8.37 (m, 12H, Ar-H), 8.18 (s, 2H, NH_2_ exchangeable with D_2_O ), 9.17 (s, 1H, CH of pyrazole); ^13^C-NMR (DMSO-*d*_6_-δ ppm): 42.3, 55.2, 55.3, 61.4, 114.0, 114.1, 114.2, 116.4, 118.9, 125.6, 126.5, 128.0, 128.9, 129.5, 131.1, 134.8, 134.8, 140.5, 150.4, 151.6, 159.2, 160.4, 175.6; MS (EI, 70 eV): *m*/*z* (%): 519 (2.9, M^+^ + 2), 517 (7, M^+^), 369 (100); Anal. Calcd. for C_27_H_24_ClN_5_O_2_S (518.03): C, 62.60; H, 4.67; N, 13.52; Found: C, 62.52; H, 4.43; N, 13.26.

#### 3.1.4. 4-(3-(4-Substituted phenyl)isoxazol-5-yl)-1-(3-chlorophenyl)-3-(4-methoxyphenyl)-1*H*-pyrazole (**5a**,**b**)

A mixture of compounds **2a**,**b** (0.01 mol) and hydroxylamine hydrochloride (0.01 mol) in ethanol (30 mL) containing sodium hydroxide solution (0.5 g NaOH in 0.5 mL water) was refluxed for 2–3 h. The reaction mixture was poured onto ice-water, neutralized with drops of concentrated Hydrochloric acid, and the solid precipitate formed filtered off, washed with water, and recrystallized from methanol to afford the desired compounds **5a**,**b**.

*4-(3-(4-Bromophenyl)isoxazol-5-yl)-1-(3-chlorophenyl)-3-(4-methoxyphenyl)-1H-pyrazole* (**5a**). Yield 85%; m.p. 168–170 °C; IR (KBr, cm^−1^) ν: 3064 (CH-Ar), 1601 (C=C); ^1^H-NMR (DMSO-*d*_6_-δ ppm): 3.77 (s, 3H, OCH_3_), 6.59–8.88 (m, 13H, Ar-H + CH-isoxazole), 9.18 (s, 1H, CH-pyrazole); ^13^C-NMR (DMSO-*d*_6_-δ ppm): 55.3, 113.7, 114.1, 114.2, 116.6, 118.6, 123.4, 126.5, 127.5, 128.6, 129.5, 129.7, 130.8, 131.3, 132.1, 134.1, 140.2, 151.5, 159.8, 164.3, 168.8; MS (EI, 70 eV): *m*/*z* (%): 509 (0.1, M^+^ + 4), 507 (0.2, M^+^ + 2), 505 (0.1, M^+^), 327 (100); Anal. Calcd. for C_25_H_17_BrClN_3_O_2_ (506.78): C, 59.25; H, 3.38; N, 8.29; Found: C, 59.48; H, 3.27; N, 8.17.

*4-(3-(4-Methoxyphenyl)isoxazol-5-yl)-1-(3-chlorophenyl)-3-(4-methoxyphenyl)-1H-pyrazole* (**5b**). Yield 80%; m.p. 227–229 °C; ^1^H-NMR (DMSO-*d*_6_-δ ppm): 3.72, 3.78 (2s, 6H, 2OCH_3_), 7.00–8.87 (m, 13H, Ar-H + CH-isoxazole ring), 9.18 (s, 1H, CH-pyrazole); MS (EI, 70 eV): *m*/*z* (%): 461 (1.5, M^+^ + 4), 459 (4.8, M^+^ + H_2_), 327 (100); Anal. Calcd. for C_26_H_20_ClN_3_O_3_ (457.91): C, 68.20; H, 4.40; N, 9.18; Found: C, 68.41; H, 4.62; N, 9.23.

#### 3.1.5. 6-(4-Substitutedphenyl)-4-(1-(3-chlorophenyl)-3-(4-methoxyphenyl)-1*H*-pyrazol-4-yl)-1,2-dihydro-2-Oxopyridine-3-carbonitrile (**6a**,**b**) and 6-(4-substitutedphenyl)-4-(1-(3-chlorophenyl)-3-(4-methoxyphenyl)-1*H*-pyrazol-4-yl)-1,2-dihydro-2-iminopyridine-3-carbonitrile (**7a**,**b**)

A mixture of compounds **2a**,**b** (0.01 mol), ethyl cyanoacetate or malononitrile (0.01 mol), and ammonium acetate (6 g, 0.08 mol) was refluxed in ethanol (30 mL) for 6 h. The formed precipitate was collected by filtration, washed several times with water, dried and recrystallized from ethanol to afford the title compounds **6a**,**b** and **7a**,**b**, respectively.

*6-(4-Bromophenyl)-4-(1-(3-chlorophenyl)-3-(4-methoxyphenyl)-1H-pyrazol-4-yl)-1,2-dihydro-2-oxopyridine-3-carbonitrile* (**6a**). Yield 75%; m.p. 217–219 °C; IR (KBr, cm^−1^) ν: 3335 (NH), 2192 (C≡N), 1687 (C=O), 1587 (C=C); ^1^H-NMR (DMSO-*d*_6_-δ ppm): 3.77 (s, 3H, OCH_3_), 6.71 (s, 1H, CH-pyridine), 6.96–8.05 (m, 13H, Ar-H + NH exchangeable with D_2_O), 9.07 (s, 1H, CH of pyrazole); ^13^C-NMR (DMSO-*d*_6_-δ ppm): 55.6, 114.7, 116.6, 117.5, 117.8, 118.7, 124.6, 125.3, 127.1, 129.4, 130.0, 130.8, 131.9, 132.4, 134.6, 140.5, 150.9, 152.3, 160.0, 162.9, 167.3; MS (EI, 70 eV): *m*/*z* (%): 558 (3.7, M^+^ + 2), 557 (1.7, M^+^ + 1), 91 (100); Anal. Calcd. for C_28_H_18_BrClN_4_O_2_ (557.83): C, 60.29; H, 3.25; N, 10.04; Found: C, 60.41; H, 3.32; N, 10.23.

*6-(4-Methoxyphenyl)-4-(1-(3-chlorophenyl)-3-(4-methoxyphenyl)-1H-pyrazol-4-yl)-1,2-dihydro-2-oxo-pyridine-3-carbonitrile* (**6b**). Yield 76%; m.p. 198–200 °C; IR (KBr, cm^−1^) ν: 3417 (NH), 2216 (C≡N), 1726 (C=O), 1654 (C=N), 1597 (C=C); ^1^H-NMR (DMSO-*d*_6_-δ ppm): 3.35 (s, 1H, NH exchangeable with D_2_O), 3.77, 3.81 (2s, 6H, 2OCH_3_), 6.60 (s, 1H, CH-pyridine), 6.97–9.00 (m, 12H, Ar-H), 9.23 (s, 1H, CH of pyrazole); ^13^C-NMR (DMSO-*d*_6_-δ ppm): 55.3, 55.5, 100.2, 114.1, 114.1, 114.5, 115.7, 115.8, 116.2, 118.2, 122.6, 124.1, 126.6, 127.7, 129.0, 130.0, 130.3, 134.1, 140.0, 150.5, 155.2, 159.5, 160.3, 161.7, 161.9; MS (EI, 70 eV): *m*/*z* (%): 510 (4, M^+^ + 2), 508 (2, M^+^), 303 (100); Anal. Calcd. for C_29_H_21_ClN_4_O_3_ (508.96): C, 68.44; H, 4.16; N, 11.01; Found: C, 68.41; H, 4.22; N, 10.88.

*6-(4-Bromophenyl)-4-(1-(3-chlorophenyl)-3-(4-methoxyphenyl)-1H-pyrazol-4-yl)-1,2-dihydro-2-imino-pyridine-3-carbonitrile* (**7a**). Yield 73%; m.p. 172–174 °C; IR (KBr, cm^−1^) ν: 3335, 3195 (2NH), 2192 (C≡N), 1587 (C=C); ^1^H-NMR (DMSO-*d*_6_-δ ppm): 3.59 (s, 1H, NH exchangeable with D_2_O), 3.78 (s, 3H, OCH_3_), 6.52 (s, 1H, CH-pyridine), 6.93–8.04 (m, 13H, Ar-H + NH exchangeable with D_2_O), 8.89 (s, 1H, CH-pyrazole); ^13^C-NMR (DMSO-*d*_6_-δ ppm): 55.6, 113.0, 114.1, 114.6, 115.5, 116.8, 118.3, 118.6, 123.1, 125.4, 126.7, 128.4, 129.3, 130.7, 131.2, 131.9, 132.1, 134.6, 140.8, 151.4, 159.8, 160.5, 164.2, 169.0; MS (EI, 70 eV): *m*/*z* (%): 507 (2.8, M^+^ + 2), 555 (7, M^+^), 149 (100); Anal. Calcd. for C_28_H_19_BrClN_5_O (556.84): C, 60.39; H, 3.44; N, 12.58; Found: C, 60.48; H, 3.27; N, 12.17.

*6-(4-Methoxyphenyl)-4-(1-(3-chlorophenyl)-3-(4-methoxyphenyl)-1H-pyrazol-4-yl)-1,2-dihydro-2-iminopyridine-3-carbonitrile* (**7b**). Yield 70%; m.p. 246–248 °C; IR (KBr, cm^−1^) ν: 3405, 3334 (2NH), 2198 (C≡N), 1573 (C=C); ^1^H-NMR (DMSO-*d*_6_-δ ppm): 3.26 (s, 1H, NH exchangeable with D_2_O), 3.74, 3.79 (2s, 6H, 2 OCH_3_), 6.92–8.05 (m, 13H, Ar-H + CH-pyridine), 7.24 (s, 1H, NH exchangeable with D_2_O), 8.88 (s, 1H, CH-pyrazole); ^13^C-NMR (DMSO-*d*_6_-δ ppm): 55.1, 55.3, 109.5, 113.9, 114.0, 114.6, 115.1, 116.4, 117.8, 118.8, 122.5, 124.5, 126.1, 128.4, 129.6, 130.0, 131.4, 134.1, 140.3, 150.0, 159.3, 160.8, 161.0, 161.5, 169.3; MS (EI, 70 eV): *m*/*z* (%): 509 (10, M^+^ + 2), 507 (14, M^+^), 55 (100); Anal. Calcd. for C_29_H_22_ClN_5_O_2_ (507.97): C, 68.57; H, 4.37; N, 13.79; Found: C, 68.41; H, 4.42; N, 13.43.

#### 3.1.6. 4-(4-Substitutedphenyl)-6-(1-(3-chlorophenyl)-3-(4-methoxyphenyl)-1*H*-pyrazol-4-yl)pyrimidin-2-amine (**8a**,**b**)

An aqueous solution of sodium hydroxide (40%, 5 mL) was added portion wise during a period of 3 h to a mixture of 1-substituted prop-2-en-1-ones **2a** or **2b** (0.01 mol) and guanidine sulphate (1.6 g, 0.01 mol) in ethanol (25 mL). After refluxing for 5–7 h, the solid product formed upon pouring onto ice/water containing a few drops of hydrochloric acid (until pH ~ 6) was collected by filtration, washed with water, then recrystallized from methanol.

*4-(4-Bromophenyl)-6-(1-(3-chlorophenyl)-3-(4-methoxyphenyl)-1H-pyrazol-4-yl)pyrimidin-2-amine* (**8a**). Yield 69%, m.p. 163–165 °C; IR (KBr, cm^−1^) ν: 3406 (NH_2_), 1581 (C=C); ^1^H-NMR (DMSO-*d*_6_-δ ppm): 3.80 (s, 3H, OCH_3_), 6.58 (s, 2H, NH_2_ exchangeable with D_2_O), 6.97–8.05 (m, 13H, Ar-H + CH-pyridine), 9.08 (s, 1H, CH-pyrazole); ^13^C-NMR (DMSO-*d*_6_-δ ppm): 55.1, 104.1, 114.0, 114.0, 116.1, 118.1, 124.0, 125.5, 126.3, 128.6, 129.9, 130.8, 131.2, 131.7, 133.9, 134.1, 140.7, 145.5, 159.4, 161.0, 162.7, 163.7; MS (EI, 70 eV): *m*/*z* (%): 535 (1.3, M^+^ + 4), 533 (7.6, M^+^ + 2), 531 (9, M^+^), 111 (100); Anal. Calcd. for C_26_H_19_BrClN_5_O (532.82): C, 58.61; H, 3.59; N, 13.14; Found: C, 58.48; H, 3.27; N, 13.17.

*4-(4-Methoxyphenyl)-6-(1-(3-chlorophenyl)-3-(4-methoxyphenyl)-1H-pyrazol-4-yl)pyrimidin-2-amine* (**8b**). Yield 71%, m.p. 224–226 °C; IR (KBr, cm^−1^) ν: 3324 (NH_2_), 1577 (C=C); ^1^H-NMR (DMSO-*d*_6_-δ ppm): 3.78, 3.81 (2s, 6H, 2 OCH_3_), 6.97 (s, 2H, NH_2_ exchangeable with D_2_O), 7.01–8.56 (m, 13H, Ar-H + CH-pyridine), 9.06 (s, 1H, CH-pyrazole; ^13^C-NMR (DMSO-*d*_6_-δ ppm): 55.3, 55.5, 103.6, 113.3, 114.0, 114.0, 115.8, 118.1, 125.2, 125.5, 126.6, 128.1, 128.9, 130.2, 131.2, 134.1, 140.7, 150.8, 160.4, 161.2, 163.5, 163.7; MS (EI, 70 eV): *m*/*z* (%): 485 (0.01, M^+^ + 2), 483 (0.02, M^+^), 135 (100); Anal. Calcd. for C_27_H_22_ClN_5_O_2_ (483.95): C, 67.01; H, 4.58; N, 14.47; Found: C, 67.41; H, 4.62; N, 14.23.

#### 3.1.7. 4-(4-Substitutedphenyl)-6-(1-(3-chlorophenyl)-3-(4-methoxyphenyl)-1*H*-pyrazol-4-yl)pyrimidine-2-(1*H*)-thione (**9a**,**b**)

A mixture of 1-substituted prop-2-en-1-ones **2a** or **2b** (0.01 mol) and thiourea (0.76 g, 0.01 mol) in ethanol (30 mL) containing (1 g, 0.025 mol) sodium hydroxide was refluxed 6–8 h. The solid product formed upon pouring onto ice/water containing a few drops of hydrochloric acid (until pH ~6) was collected by filtration, washed with water, then recrystallized from methanol to yield the desired compounds **9a**,**b**.

*4-(4-Bromophenyl)-6-(1-(3-chlorophenyl)-3-(4-methoxyphenyl)-1H-pyrazol-4-yl)pyrimidine-2-(1H)-thione* (**9a**). Yield 81%; m.p. 180–182 °C; IR (KBr, cm^−1^) ν: 3384 (NH), 1591 (C=C), 1174 (C=S); ^1^H-NMR (DMSO-*d*_6_-δ ppm): 3.77 (s, 3H, OCH_3_), 5.23 (s, 1H, NH exchangeable with D_2_O), 7.00–7.85 (m, 13H, Ar-H + CH-thiopyrimidine), 9.25 (s, 1H, CH-pyrazole); ^13^C-NMR (DMSO-*d*_6_-δ ppm): 55.2, 113.4, 114.0, 116.3, 117.1, 118.3, 122.0, 125.5, 126.8, 128.6, 129.2, 130.2, 131.1, 131.7, 134.0, 134.1, 140.6, 150.4, 160.8, 164.4, 176.1, 181.1; MS (EI, 70 eV): *m*/*z* (%); MS *m*/*z* (%): 535 (1.3, M^+^ + 4), 533 (7.6, M^+^ + 2), 531 (9, M^+^), 155 (100); Anal. Calcd. for C_26_H_18_BrClN_4_OS (549.87): C, 56.79; H, 3.30; N, 10.19; Found: C, 56.48; H, 3.27; N, 10.17.

*4-(4-Methoxyphenyl)-6-(1-(3-chlorophenyl)-3-(4-methoxyphenyl)-1H-pyrazol-4-yl)pyrimidine-2-(1)-thione* (**9b**). Yield 80%; m.p. 233–235 °C; IR (KBr, cm^−1^) ν: 3384 (NH), 1591 (C=C), 1174 (C=S); ^1^H-NMR (DMSO-*d*_6_-δ ppm): 3.78 (br s, 6H, 2 OCH_3_), 7.00–7.85 (m, 14H, Ar-H, CH-thiopyrimidine and NH exchangeable with D_2_O), 9.26 (s, 1H, CH-pyrazole); ^13^C-NMR (DMSO-*d*_6_-δ ppm): 55.2, 55.7, 101.9, 109.4, 114.0, 114.2, 115.0, 116.3, 117.9, 125.2, 125.8, 126.6, 127.4, 128.5, 130.0, 130.8, 135.1, 150.1, 159.7, 161.0, 165.1, 176.3, 180.3; MS (EI, 70 eV): *m*/*z* (%); MS *m*/*z* (%): 563 (0.7, M^+^ + 2), 561 (1.6, M^+^), 310 (100); Anal. Calcd. for C_27_H_21_ClN_4_O_2_S (501): C, 64.73; H, 4.22; N, 11.18; Found: C, 64.41; H, 4.62; N, 11.23.

### 3.2. Measurement of Anti-Inflammatory Activity

Compounds **2**–**9** were screened for their in vivo anti-inflammatory activity by the carrageenan-induced paw edema standard method [1]. Mature Swiss male albino rats were obtained from The Animal House, NRC, Cairo, weighing 150–200 g. Edema was induced in the left hind paw of all rats by subcutaneous injection of 0.1 mL of 1% (*w*/*v*) carrageenan in distilled water into their footpads. Rats were divided into six groups of six rats each. The first group was kept as control, and was given the respective volume of the solvent (1% of tween-80 in distilled water). The other groups were orally administered the drugs—indomethacin and celebrex (reference standards) and the tested compoundsafter dissolution in water and 1% tween 80 in dose of 0.28 mmol/kg, 1 h before carrageenan injection. The paw volume of each rat was measured using Vernier caliper; before carrageenan injection and then hourly for 4 h post-administration of the drugs. The edema rate and inhibition rate of each group were calculated as follows:

Percentage change of Edema rate (E) % = [(Vt − Vo )/Vo] × 100

Inhibition rate (I)% = [(Ec − Et)/Ec] × 100

where: Vo is the volume before carrageenan injection (mL). Vt is the volume at t hour after carrageenan injection (mL). Ec is the edema rate of control group. Et is the edema rate of the treated group.

### 3.3. Ulcerogenic Liability

After five hours of measuring the anti-inflammatory activity, the rats were sacrificed by decapitation. Their stomachs were removed, opened along the greater culvature, and the number of ulcers was assessed by the reported standard method [2]. The separate groups which received indomethacin and Celebrex (0.28 mmol/kg) as positive controls were used. The results were compared with tween-80 (1% solution) treated group as negative control.

### 3.4. Molecular Modeling Study

All the molecular modeling calculations and docking simulation studies were performed using Molecular Operating Environment (MOE^®^) [3] 2008.10. All the interaction energies and different calculations were automatically calculated.

#### 3.4.1. Optimization of the Target Compound **6b**

The target compound **6b** was constructed into a 3D model using the builder interface of the MOE program. After checking their structures and the formal charges on atoms by 2D depiction, the following steps were carried out: the target compound was subjected to a conformational search. All conformers were subjected to energy minimization, all the minimizations were performed with MOE until a RMSD gradient of 0.01 Kcal/mole and RMS distance of 0.1 Å with MMFF94X force-field and the partial charges were automatically calculated. The obtained database was then saved as MDB file to be used in the docking calculations.

#### 3.4.2. Optimization of the Enzymes Active Site

The X-ray crystallographic structure of COX-2 receptor complexed with 1-phenylsulfona-mide-3-trifluoromethyl-5-(4-bromophenyl)pyrazole, SC-558 (PDB ID: 1CX2) [4] was obtained from the Protein Data Bank through the internet. The enzyme was prepared for docking studies by removing the ligand molecule SC-558 from the COX-2 receptor active site. Hydrogen atoms were added to the system with their standard geometry. The atoms connection and type were checked for any errors with automatic correction. Selection of the receptor and its atom potential were fixed. MOE Alpha Site Finder was used for the active site search in the enzyme structure using all default items. Dummy atoms were created from the obtained alpha spheres. Re-docking of co-crystalline ligand to the receptor active site to insure the docking method was efficient and the active pocket was saved as a MOE file to be used for docking simulation of the selected compounds.

#### 3.4.3. Docking of the Target Molecule **6b** and Celecoxib to the Receptor Active Sites

Docking of the conformation database of the target compounds was done using MOE-Dock software. The following methodology was generally applied via loading of the enzyme active site file and the dock tool was initiated. The program specifications were adjusted to:

- Dummy atoms as the docking site.
- Triangle matcher as the placement methodology to be used.
- London dG as scoring methodology to be used and was adjusted to its default values.

The MDB file of the ligand to be docked was loaded and dock calculations were run automatically. The obtained poses were studied and the poses showed best ligand-enzyme interactions were selected and stored for energy calculations. The 2D interaction and stereo view for compound **6b** inside the active site of COX-2 kinase were obtained and saved as both MOE and photo files.

## 4. Conclusions

In summary, we have designed and synthesized a new series of 1,3,-diaryl pyrazole derivatives linked different nitrogenous heterocyclic ring systems at C-4 position including pyrazoles, isoxazole, pyridines, or pyrimidines and evaluated for their anti-inflammatory activity using standard acute carrageenan-induced paw edema method. From the obtained results, six compounds (**2a**, **2b**, **3a**, **6b**, **7b**, and **9b**) showed consistently excellent anti-inflammatory activity (84.39–89.57% inhibition) 4 h after the carrageenan injection comparable to that of the standard drugs indomethacin and Celebrex (72.99% and 83.76%, respectively). The cyanopyridone derivative **6b** seems to be the most effective product, displayed better activity (89.57% inhibition of edema) than both indomethacin and celecoxib (reference standards), and could be considered a promising selective anti-inflammatory lead for further development of more potent anticancer agents. The structures of the newly prepared compounds were elucidated using spectroscopic and elemental analysis.

## Figures and Tables

**Table 1:** Anti-inflammatory activity of the tested compounds **2**–**9** using carrageenan-induced paw edema in rats.

Drugs	%Change	% Inhibition	%Change	% Inhibition	%Change	% Inhibition	%Change	% Inhibition
	1 h		2 h		3 h		4 h	
**control**	100.5 ± 9.07	----	113.8 ± 13.21	----	102.4 ± 10.42	----	32.05 ± 2.80	----
**2a**	88.78 ± 9.70	−11.62	94.47 ± 10.13	−17.00	58.42 ± 7.31 *	−42.93	4.735 ± 1.92 *	−85.23
**2b**	77.43 ± 2.25	−22.92	82.37 ± 2.55	−27.62	36.27 ± 3.73 *	−64.57	4.557 ± 0.99 *	−85.78
**3a**	64.03 ± 4.96 *	−36.26	70.22 ± 5.50 *	−38.30	63.92 ± 3.59 *	−37.56	5.003 ± 0.94 *	−84.39
**3b**	91.8 ± 9.65	−8.61	98.19 ± 9.24	−13.72	48.54 ± 4.24 *	−52.58	19.56 ± 2.79	−38.96
**4a**	96.39 ± 6.90	−4.05	103 ± 6.95	−9.51	131 ± 12.23	27.98	35.83 ± 1.50	11.80
**4b**	55.82 ± 4.05 *	−44.43	64.57 ± 5.18 *	−43.27	28.38 ± 2.86 *	−72.28	7.442 ± 1.59 *	−76.78
**5a**	73.41 ± 5.92	−26.93	78.32 ± 7.00	−31.18	66.51 ± 6.60 *	−35.02	19.73 ± 2.44	−38.45
**5b**	100.4 ± 10.72	−0.06	107.8 ± 10.97	−5.26	53.77 ± 5.47 *	−47.47	10.38 ± 1.77 *	−67.62
**6a**	86.3 ± 2.17	−14.09	93.78 ± 2.51	−17.60	84.33 ± 6.57	−17.62	44.02 ± 5.39	37.35
**6b**	41.96 ± 3.82 *	−58.23	61.37 ± 4.23 *	−46.08	53.86 ± 4.94 *	−47.39	3.345 ± 0.52 *	−89.57
**7a**	84.47 ± 4.65	−15.92	86.53 ± 2.72	−23.97	114.6 ± 6.95	12.00	38.18 ± 5.73	19.11
**7b**	74.78 ± 5.38	−25.56	80.48 ± 5.51	−29.28	61.94 ± 3.79 *	−39.49	4.368 ± 1.18 *	−86.37
**8a**	60.3 ± 4.08 *	−39.97	71.13 ± 6.50 *	−37.50	82.85 ± 8.84	−19.07	26.39 ± 2.92	−17.66
**8b**	77.38 ± 6.13	−22.97	112.4 ± 10.61	−1.25	76.74 ± 6.85	−25.03	18.34 ± 2.40 *	−42.78
**9a**	52.79 ± 5.44 *	−47.45	61.83 ± 6.32 *	−45.67	68.91 ± 5.30	−32.68	22.56 ± 0.81	−29.61
**9b**	39.06 ± 2.75 *	−61.11	46.06 ± 3.05 *	−59.53	62.15 ± 6.83 *	−39.29	4.033 ± 0.87 *	−87.42
**Indomethacin**	60.14 ± 6.64 *	−40.1295	67.97 ± 6.03 *	−40.2745	62.27 ± 8.14 *	−39.17	8.655 ± 1.53 *	−72.9943
**Celebrex**	52.39 ± 3.03 *	−47.8509	58.69 ± 2.95 *	−48.4341	60.25 ± 5.74 *	−41.1372	5.205 ± 0.65 *	−83.7597

Values represent the mean ± S.E. of six animals for each groups. * *p* < 0.05: Statistically significant from the control using one-way ANOVA (using Tukey as post hoc test).

**Table 2:** Docking results of the compounds **2**–**9** with COX-2 enzyme in comparison with the ligands, SC-558 and celecoxib using MOE software version 2008.10.

Compd. NO.	Docking Score (Kcal/mol)	Amino Acid Residues (Bond Length Å)	Atoms of Compound	Type of Bond
**2a**	−6.78	His90 (2.6);	O(CO)	H-acc
		Arg120 (2.1);	O(OCH_3_) (parent)	H-acc
**2b**	−6.93	His90 (2.4);	O(CO)	H-accH-accArene-cation Arene-cation
		Arg120 (2.3);	O(OCH_3_) (parent)	
		Arg120;	C_6_H_3_-4-OCH_3_ (new)	
		Arg513	C_6_H_3_-4-OCH_3_ (new)	
**3a**	−7.12	Arg120 (1.8);	O(OCH_3_) (parent)	H-acc
**3b**	−5.46	Arg120 (2.1);	O(OCH_3_) (parent)	H-accArene-cation Arene-cation
		Arg120;	C_6_H_3_-4-OCH_3_ (new)	
		Arg513	C_6_H_3_-4-OCH_3_ (new)	
**4a**	−5.22	Arg120 (2.6);	O(OCH_3_) (parent)	H-acc
**4b**	−7.24	Arg120 (2.4);	O(OCH_3_) (parent)	H-accArene-cation Arene-cation
		Arg120;	C_6_H_3_-4-OCH_3_ (new)	
		Arg513	C_6_H_3_-4-OCH_3_ (new)	
**5a**	−5.14	Arg120 (2.7);	O(OCH_3_) (parent)	H-acc
**5b**	−6.25	Arg120 (2.1);	O(OCH_3_) (parent)	H-acc Arene-cation Arene-cation
		Arg120;	C_6_H_3_-4-OCH_3_ (new)	
		Arg513	C_6_H_3_-4-OCH_3_ (new)	
**6a**	−5.43	His90 (2.4);	NH(pyridone)	H-acc
		His90 (2.6);	O(pyridone)	H-acc
		Arg120 (2.5);	O(OCH_3_) (parent)	H-acc
**6b**	−8.11	His90 (2.7);	NH(pyridone)	H-accH-accH-accArene-cation Arene-cation
		His90 (2.7);	O(pyridone)	
		Arg120 (3.1);	O(OCH_3_) (parent)	
		Arg120;	C_6_H_3_-4-OCH_3_ (new)	
		Arg513	C_6_H_3_-4-OCH_3_ (new)	
**7a**	−5.45	His90 (2.6);	NH(iminopyridine)	H-accH-acc
		Arg120 (2.3);	O(OCH_3_) (parent)	
**7b**	−6.98	His90 (2.7);	NH(iminopyridine)	H-accH-accArene-cation Arene-cation
		Arg120 (2.1);	O(OCH_3_) (parent)	
		Arg120;	C_6_H_3_-4-OCH_3_ (new)	
		Arg513	C_6_H_3_-4-OCH_3_ (new)	
**8a**	−5.20	Arg120 (2.2);	O(OCH_3_) (parent)	H-acc
**8b**	−6.26	Arg120 (1.9);	O(OCH_3_) (parent)	H-accArene-cation Arene-cation
		Arg120;	C_6_H_3_-4-OCH_3_ (new)	
		Arg513	C_6_H_3_-4-OCH_3_ (new)	
**9a**	−5.75	Arg120 (2.5);	O(OCH_3_) (parent)	H-acc
**9b**	−7.48	Arg120 (1.9);	O(OCH_3_) (parent)	H-accArene-cation Arene-cation
		Arg120;	C_6_H_3_-4-OCH_3_ (new)	
		Arg513	C_6_H_3_-4-OCH_3_ (new)	
**SC-558**	−6.30	His90 (2.2);	H(NH_2_)	H-acc
		GIn192 (2.4);	H(NH_2_)	H-don
		Arg513 (2.2);	H(NH_2_)	H-don
		Val523;	C_6_H_3_-4-Br	Arene-Arene
		Ala527;	C_6_H_3_-4-Br	Arene-Arene
		Leu352;	Phenylsulphonamide	Arene-Arene
		Ser353	Phenylsulphonamide	Arene-Arene
**Celecoxib**	−6.55	His90 (1.9);	H(NH_2_)	H-acc
		GIn192 (2.1);	H(NH_2_)	H-don
		Leu338 (2.3);	H(NH_2_)	H-acc
		Val523;	C_6_H_3_-4-OCH_3_	Arene-Arene
		Ala527;	C_6_H_3_-4-OCH_3_	Arene-Arene
		Leu352;	Phenylsulphonamide	Arene-Arene
		Ser353	Phenylsulphonamide	Arene-Arene
